# Comparison of Trends in Tuberculosis Incidence among Adults Living with HIV and Adults without HIV – Kenya, 1998–2012

**DOI:** 10.1371/journal.pone.0099880

**Published:** 2014-06-17

**Authors:** Courtney M. Yuen, Herman O. Weyenga, Andrea A. Kim, Timothy Malika, Hellen Muttai, Abraham Katana, Lucy Nganga, Kevin P. Cain, Kevin M. De Cock

**Affiliations:** 1 Epidemic Intelligence Service, assigned to Division of Tuberculosis Elimination, Centers for Disease Control and Prevention, Atlanta, Georgia, United States of America; 2 Division of Leprosy, Tuberculosis and Lung Disease, Ministry of Health, Nairobi, Kenya; 3 Centers for Disease Control and Prevention Kenya, Nairobi, Kenya; UCL Institute of Child Health, University College London, United Kingdom

## Abstract

**Background:**

In Kenya, the comparative incidences of tuberculosis among persons with and without HIV have not been described, and the differential impact of public health interventions on tuberculosis incidence in the two groups is unknown.

**Methods:**

We estimated annual tuberculosis incidence stratified by HIV status during 2006–2012 based on the numbers of reported tuberculosis patients with and without HIV infection, the prevalence of HIV infection in the general population, and the total population. We also made crude estimates of annual tuberculosis incidence stratified by HIV status during 1998–2012 by assuming a constant ratio of HIV prevalence among tuberculosis patients compared to the general population.

**Results:**

Tuberculosis incidence among both adults with HIV and adults without HIV increased during 1998–2004 then remained relatively stable until 2007. During 2007–2012, tuberculosis incidence declined by 28–44% among adults with HIV and by 11–26% among adults without HIV, concurrent with an increase in antiretroviral therapy uptake. In 2012, tuberculosis incidence among adults with HIV (1,839–1,936 cases/100,000 population) was still eight times as high as among adults without HIV (231–238 cases/100,000 population), and approximately one third of tuberculosis cases were attributable to HIV.

**Conclusions:**

Although tuberculosis incidence has declined among adults with and without HIV, the persistent high incidence of tuberculosis among those with HIV and the disparity between the two groups are concerning. Early diagnosis of HIV, early initiation of antiretroviral therapy, regular screening for tuberculosis, and isoniazid preventive therapy among persons with HIV, as well as tuberculosis control in the general population, are required to address these issues.

## Introduction

Despite declining incidence in recent years, tuberculosis remains a leading cause of death among infectious diseases globally, second only to HIV [1]. In addition, the concurrent global tuberculosis and HIV epidemics interact synergistically, with HIV increasing tuberculosis incidence and tuberculosis being associated with both mortality and incidence of other AIDS-defining illnesses among persons living with HIV [2].

HIV is an important driver of the tuberculosis epidemic in Kenya, which is classified as a high-burden country for both tuberculosis and HIV. In 2012, 39% of all reported tuberculosis cases in Kenya occurred in persons living with HIV [1]. Studies from Kenya have reported HIV infection to be associated with default during tuberculosis treatment [3] and increased mortality in tuberculosis patients [4]. However, while HIV test results for newly diagnosed tuberculosis cases have been recorded in Kenya since 2006, the comparative incidences of tuberculosis among persons living with HIV and persons without HIV have not been described. As a result, the differential impact of public health interventions on tuberculosis incidence in the two groups is unknown.

Provision of antiretroviral therapy and tuberculosis preventive therapy to persons living with HIV have been shown to substantially reduce tuberculosis incidence in this population [5]–[8]. In 2004, Kenya began a scale-up of antiretroviral therapy in the public sector and started to integrate HIV testing and care into the services provided for tuberculosis patients [9]. Current Kenyan guidelines recommend that persons living with HIV be screened for tuberculosis, and that those diagnosed with tuberculosis be started on tuberculosis therapy. However, inadequate data exist to determine the quality of this screening and diagnosis process. In 2011, only 65% of persons living with HIV who also had newly diagnosed tuberculosis received treatment for both HIV and tuberculosis [10]. Furthermore, tuberculosis preventive therapy has not yet been widely implemented in Kenya [10].

Comparing tuberculosis incidence among persons with and without HIV can uncover distinct trends in the epidemiology of tuberculosis in these two populations [11] and can inform policy on the specific interventions needed, as well as on the prioritization of tuberculosis control and prevention efforts among persons living with HIV. To help guide future programmatic interventions aimed at reducing tuberculosis incidence and to gain insight into the differential impact of existing interventions on people with and without HIV, we estimated annual tuberculosis incidence stratified by HIV status and annual population attributable fraction of tuberculosis due to HIV.

## Methods

### Detailed Estimate of Tuberculosis Incidence Stratified by HIV Status

We estimated annual tuberculosis incidence among adults living with HIV and adults without HIV in Kenya during 2006–2012 based on the numbers of reported tuberculosis patients with and without HIV infection, the prevalence of HIV infection in the general population, and the total population. Because our data source for prevalence of HIV infection was limited to adults aged 15–64 years, we focused our analysis on this age group. We determined the population attributable fraction of tuberculosis cases due to HIV by first calculating the difference between overall tuberculosis incidence and estimated incidence of tuberculosis among persons without HIV, then dividing this value by the overall tuberculosis incidence.

We used data from the 2007 and 2012 Kenya AIDS Indicator Surveys (KAIS) [12], [13] to estimate prevalence of HIV infection among adults aged 15–64 by year during 2006–2012. We assumed a constant rate of change to estimate prevalence of HIV infection during the years before and between surveys. We obtained populations, by age, from the 1999 and 2009 Kenya censuses. We estimated populations during years between and after the censuses assuming a constant annual percentage growth in the total population and applying proportions to estimate the population of adults aged 15–64 years. For each year, we applied the estimated prevalence of HIV infection to the estimated population of adults to obtain populations of adults aged 15–64 with and without HIV infection.

We obtained numbers of new tuberculosis cases reported each year in adults during 2006–2012 from the Kenya Ministry of Health Division of Leprosy, Tuberculosis and Lung Disease (DLTLD). Because case counts were disaggregated only by pediatric (age <15 years) and adult (age ≥15 years) groups, we were unable to exclude patients aged >64 years. We therefore included all adult cases in our analysis and assumed them to all be aged under 64 years. We also performed a sensitivity analysis in which we instead assumed 95% of tuberculosis cases among adults to have occurred in those aged 15–64 years, based on the age distribution of adult tuberculosis patients in select provinces for which disaggregated data were available. For each year, we obtained the numbers of adult tuberculosis patients who had HIV test results and the number with HIV-positive results.

We made upper-bound estimates for the total number of adult tuberculosis patients with HIV by assuming the HIV prevalence in patients without HIV test results to be the same as in patients with HIV test results. We made lower-bound estimates by assuming the prevalence of HIV infection in patients without HIV test results to be the same as in the general adult population.

### Trends in Antiretroviral Therapy Uptake

We obtained data on the numbers of adults in Kenya receiving antiretroviral therapy, by year, from the National AIDS and STI Control Program. We assumed that the number of adults aged >64 years receiving antiretroviral therapy was negligible. We calculated antiretroviral therapy uptake among adults (i.e., the proportion of adults living with HIV who are receiving antiretroviral therapy) each year during 2006–2012 using the estimated population of adults aged 15–64 living with HIV in Kenya (method described above). This calculation of antiretroviral therapy uptake reflects the overall proportion of people living with HIV on therapy, and does not describe uptake according to national guidelines, nor the effect of changes in guidelines over the study period.

### Crude Estimate of Tuberculosis Incidence Stratified by HIV Status

To evaluate trends over a longer time period than the years for which availability of HIV test results for tuberculosis patients would allow, we obtained number of adult tuberculosis cases by year from 1998, the first year when the estimated case detection rate exceeded 70% [14], through 2012. We used the UNAIDS Estimates and Projection Package (EPP) model to estimate the annual prevalence of HIV infection in the adult population during this time period [15]. We assumed an HIV prevalence ratio of six for tuberculosis patients compared to the general population because this ratio caused the crude estimates for 2006–2012 to be consistent with estimates from our detailed analysis, and because this was the average ratio observed across six groups of developing countries in an analysis of the global burden of tuberculosis attributable to HIV [16].

### Data Availability Statement

All data used in this manuscript represent publically available information. Annual numbers of reported tuberculosis cases, HIV test results for tuberculosis patients, and numbers of adults receiving antiretroviral therapy are available by request from the Kenya Ministry of Health (P.O. Box 30016, Nairobi, Kenya; telephone: +254-20-717077; www.health.go.ke). Data from the 2007 and 2012 KAIS have been published [12], [13]. Population estimates are available by request from the Kenya National Bureau of Statistics (P.O. Box 30266-00100, Nairobi; telephone: +254-20-317583; www.knbs.or.ke).

### Ethics Statement

The project was determined not to be human subjects research by the U.S. Centers for Disease Control and Prevention (CDC) and did not require approval by an institutional review board.

## Results

### Detailed Estimate of Tuberculosis Incidence, by HIV Status, 2006–2012

**Figure 1:**
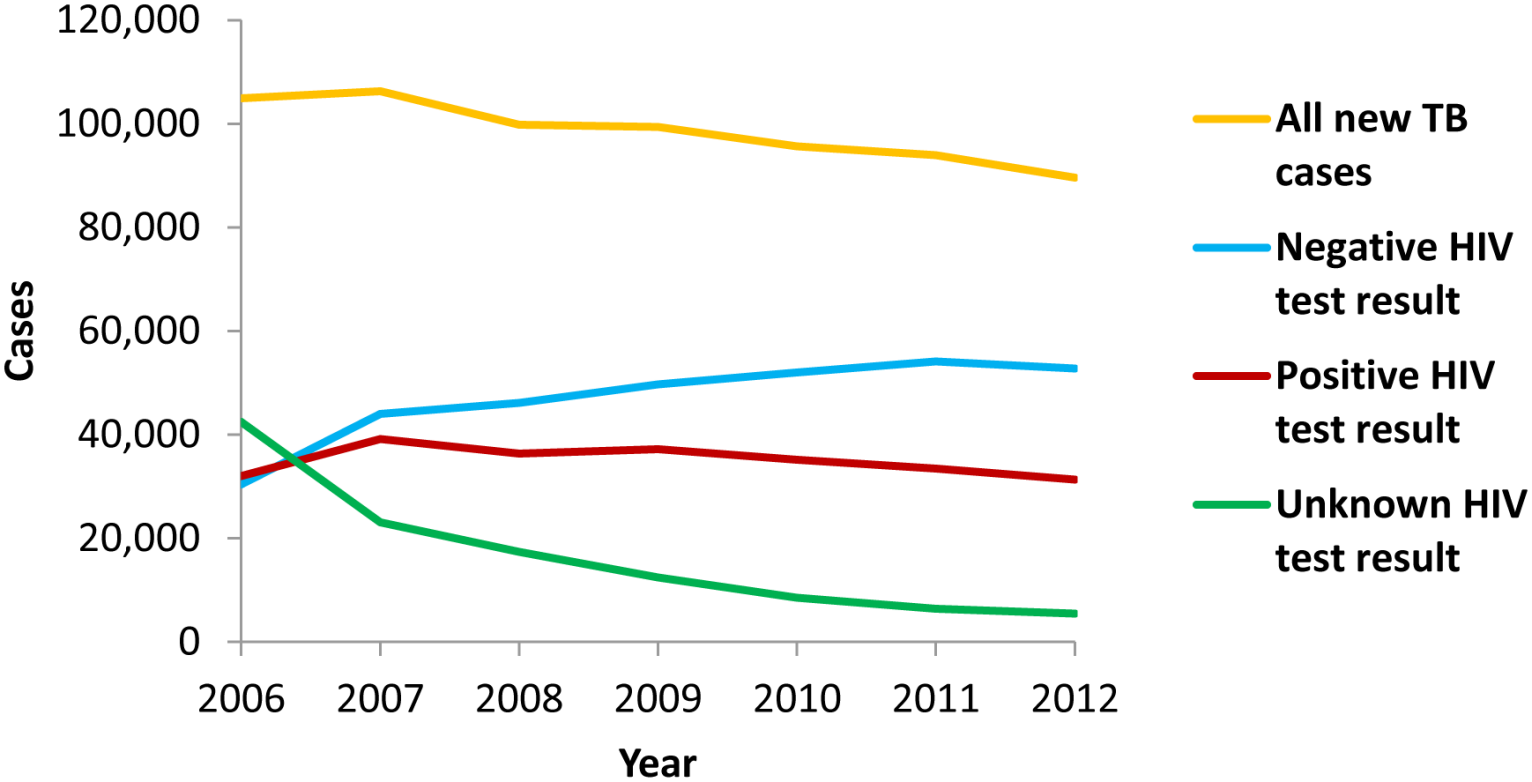
New tuberculosis cases, by HIV test result – Kenya, 2006–2012. Total numbers of new tuberculosis cases (orange line), new tuberculosis cases in patients with negative HIV test result (blue line), new tuberculosis cases in patients with positive HIV test result (red line), and new tuberculosis cases in patients with unknown HIV test result (green line).

The number of new tuberculosis cases reported by year in Kenya during 2006–2012 is shown in Table 1 and Figure 1. The proportion of tuberculosis patients with HIV test results increased from 59.5% in 2006 to 93.9% in 2012 (Table 1). The proportion of patients with positive HIV test results among those tested decreased from 51.3% in 2006 to 37.2% in 2012 (Table 1). The number of patients with positive HIV test results also decreased during 2007–2012 (Figure 1). Based on the results of KAIS, the prevalence of HIV infection among adults aged 15–64 years was estimated to be 7.1% (95% confidence interval [CI]: 6.5–7.7) in 2007 and 5.6% (95% CI: 4.9–6.3) in 2012 [12], [13].

**Table 1:** Number and HIV test results of new tuberculosis patients –Kenya, 2006–2012.

Year	New tuberculosis patients	Proportion with HIV test result	Proportion with positive result among those tested
2006	104,935	59.50%	51.30%
2007	106,261	78.30%	47.10%
2008	99,807	82.60%	44.00%
2009	99,354	87.50%	42.80%
2010	95,604	91.10%	40.30%
2011	93,964	93.20%	38.20%
2012	89,143	93.90%	37.20%

**Figure 2:**
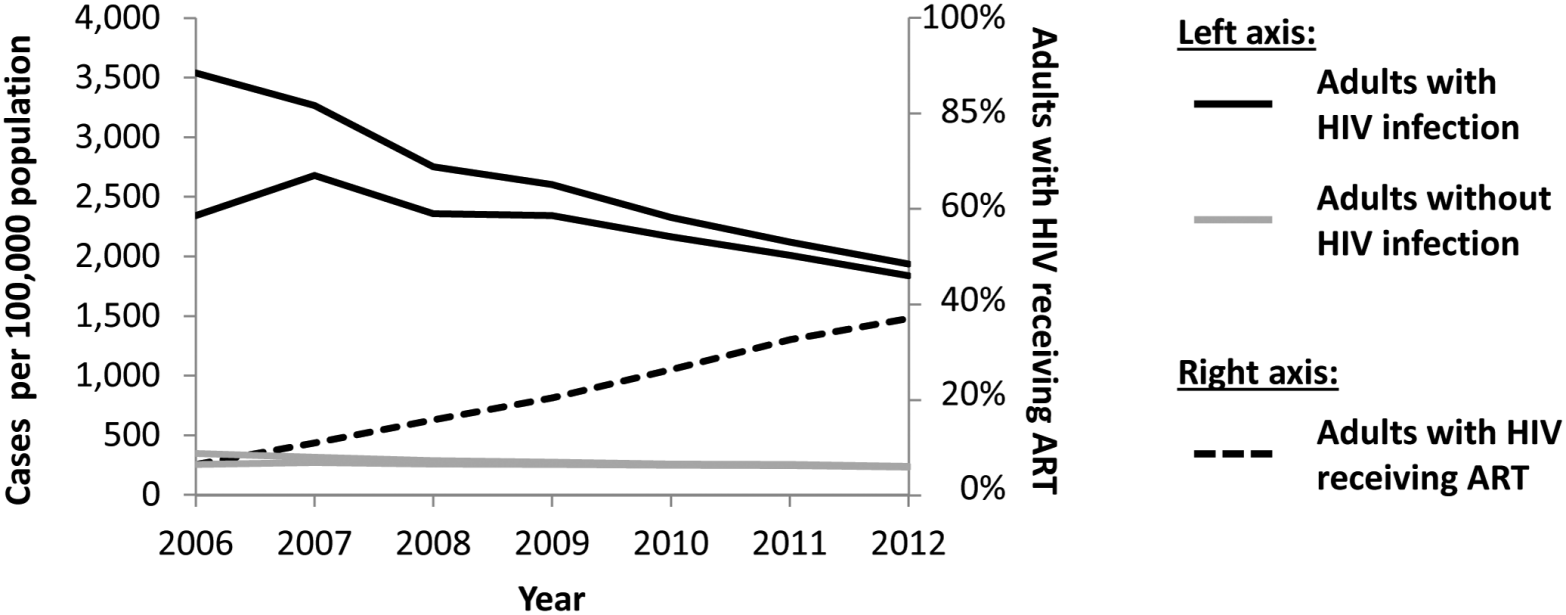
Tuberculosis incidence estimates and antiretroviral therapy uptake among adults – Kenya, 2006–2012. Upper- and lower-bound estimates of tuberculosis incidence among adults aged 15–64 years with (solid black line) and without (solid grey line) HIV infection, and proportion of adults aged 15–64 years with HIV infection who were receiving antiretroviral therapy (ART) (dashed black line).

Figure 2 shows estimated tuberculosis incidence during 2006–2012 among adults aged 15–64 years, by HIV status. Because of the substantial proportion of patients in 2006 without HIV test results available, it is unclear how tuberculosis incidence changed during 2006–2007. However, during 2007–2012, tuberculosis incidence declined by 28–44% among adults living with HIV and by 11–26% among adults without HIV. In 2012, tuberculosis incidence among adults living with HIV was 1,839–1,936 cases per 100,000 population, while tuberculosis incidence among adults without HIV was 231–238 cases per 100,000 population. During 2006–2012, the estimated overall proportion of adults living with HIV who were receiving antiretroviral therapy increased from 7% to 37% (Figure 2).

**Figure 3:**
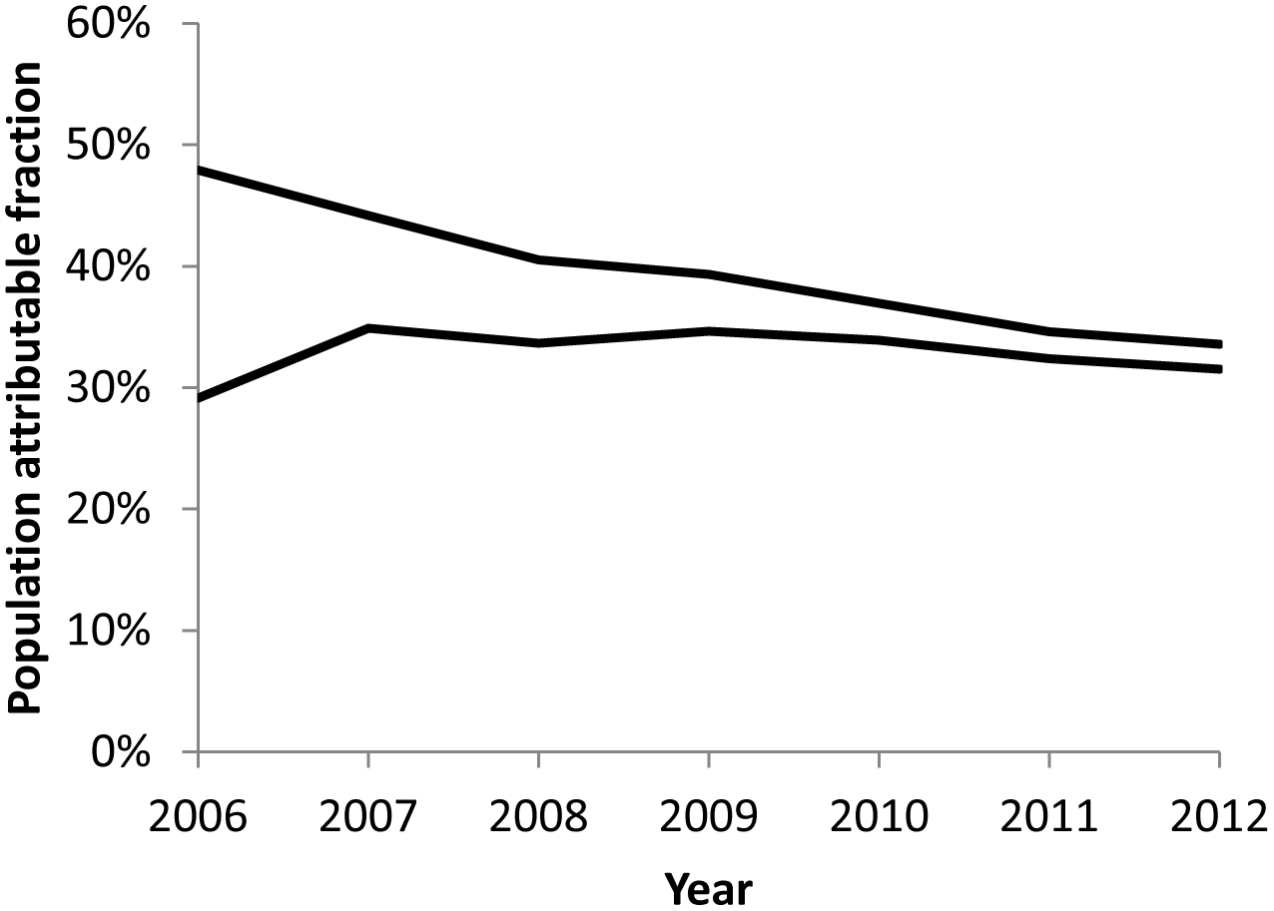
Estimates of tuberculosis attributable to HIV among adults – Kenya, 2006–2012. Upper- and lower-bound estimates of population attributable fraction of incident tuberculosis attributable to HIV among adults aged 15–64 years.

In 2012, 32–34% of tuberculosis cases among adults aged 15–64 years in Kenya were attributable to HIV (Figure 3). However, because of the large difference between upper- and lower-bound incidence estimates for the early years of the analysis period, it is unclear how the population attributable fraction has changed during 2006–2012.

To assess the robustness of our estimate, we performed sensitivity analyses to determine how making different assumptions about the age distribution of tuberculosis patients would change our results. Rather than assuming a negligible number of tuberculosis patients over 64 years of age, we assumed 95% of tuberculosis cases among adults to have occurred in those aged 15–64 years, based on the age distribution of adult tuberculosis patients in select regions for which disaggregated data were available. We then tested two assumptions for the age distribution of positive HIV test results: either that all positive HIV test results in adults occurred among those aged 15–64 years or that the proportion of adult tuberculosis patients with positive HIV test results was the same among those aged 15–64 years as among those over aged 64 years. Using either set of assumptions changed our estimates by less than 10%, and overall trends in tuberculosis incidence during the analysis period remained the same.

### Crude Estimate of Tuberculosis Incidence, by HIV Status, 1998–2012

**Figure 4:**
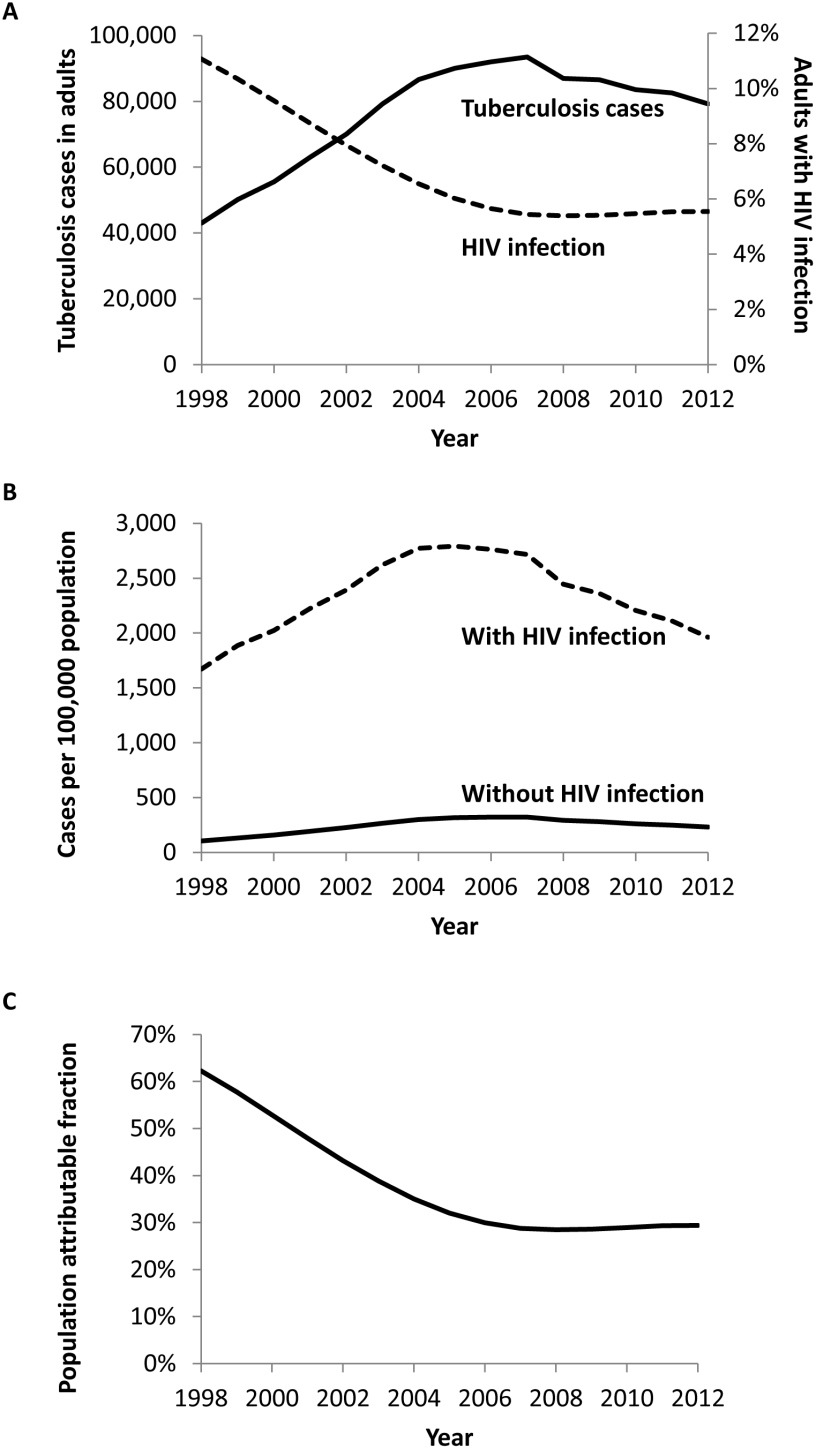
Crude estimates of tuberculosis incidence among adults with and without HIV infection – Kenya, 1998–2012. Adults are defined as persons ≥15 years of age. (A) Reported tuberculosis cases in adults (solid line) and estimated prevalence of HIV infection in the general adult population (dashed line), (B) estimated tuberculosis incidence among adults with (dashed line) and without (solid line) HIV infection, (C) estimated population attributable fraction of tuberculosis cases attributable to HIV among adults.

The number of reported tuberculosis cases in Kenya and the estimated prevalence of HIV infection in adults based on the EPP model [15] during 1998–2007 are shown in Figure 4A. Crude estimates of tuberculosis incidence based on an assumed HIV prevalence ratio of six for tuberculosis patients compared to the general population are shown in Figure 4B. Among persons living with HIV, tuberculosis incidence increased during 1998–2004, remained relatively stable until 2007 at approximately 2,750 cases per 100,000 population, then declined to 1,962 cases per 100,000 population in 2012. Among persons without HIV, the trend was similar, but overall incidence was substantially lower, peaking at approximately 320 cases per 100,000 population during 2005–2007, and declining to 231 cases per 100,000 population in 2012. Based on these estimates, the population attributable fraction of tuberculosis due to HIV declined steadily from 62% in 1998 to 29% in 2007, then remained stable (Figure 4C).

## Discussion

Using reported HIV test results among tuberculosis patients and estimates of HIV prevalence from population-based surveys, we estimated tuberculosis incidence among adults in Kenya, stratified by HIV status. Tuberculosis incidence among adults living with HIV and among adults without HIV both appear to have declined since 2007. However, despite success in the scale-up of antiretroviral therapy in Kenya, tuberculosis incidence among adults living with HIV in 2012 was still nearly 2,000 per 100,000 population, roughly eight times as high as among adults without HIV. Furthermore, during 2006–2012, over 30% of new adult tuberculosis cases were attributable to HIV while the prevalence of HIV infection in the adult population ranged from 6–7%, suggesting the disproportionate burden of tuberculosis borne by persons living with HIV.

With an overall tuberculosis incidence of 272 per 100,000 population in 2012, the annual rate of decline of less than 6% observed over the past three years in Kenya [1] is insufficient for the country to achieve the global target of eliminating tuberculosis as a public health problem by 2050. To accelerate the decline in tuberculosis incidence, a combination of interventions will be necessary. Mathematical models suggest that on a global scale, improving diagnosis and treatment of active disease, the primary focus of most national tuberculosis programs, could at best bring about an 8.5-fold decrease in tuberculosis incidence by 2050 [17]. Achieving tuberculosis elimination, defined as an incidence of 1 case per million population, by 2050 will require not only increasing tuberculosis case detection but also implementing good measures to prevent tuberculosis, including treatment of latent infection [17]. In countries with high burdens of HIV, tuberculosis prevention among persons living with HIV will require providing both antiretroviral therapy to prevent development of active disease and tuberculosis preventive therapy to treat latent infection [17], [18].

Antiretroviral therapy has been shown to substantially decrease tuberculosis incidence in persons living with HIV, particularly when it is initiated while CD4 counts are still comparatively high [5], [6], [19]. Consistent with this effect, we observed that the scale-up in antiretroviral therapy programs in Kenya coincided with a more pronounced decline in tuberculosis incidence during 2007–2012 among adults with HIV than among adults without HIV, although we cannot conclude causality based on our analysis. Specific interventions that may have contributed to the decrease in tuberculosis incidence among persons with HIV during this period include raising the CD4 threshold for initiation on antiretroviral therapy and expanding HIV testing services. The CD4 threshold defining general eligibility for initiation of antiretroviral therapy in adults was raised from 200 cells/mm^3^ to 250 cells/mm^3^ in 2007, and to 350 cells/mm^3^ in 2010 [10]. Furthermore, the availability of HIV testing services was increased from three sites in 1999 to nearly 2,000 sites in 2010, and provider-initiated testing policies were introduced in addition to existing voluntary testing services [10], [20].

However, while the scale-up of antiretroviral therapy programs in Kenya over the past several years has been impressive, almost two thirds of adults living with HIV in Kenya were not receiving antiretroviral therapy in 2012 [13]. Of these, 26% were unaware of their HIV status, and 66% were aware of their HIV status but not eligible for antiretroviral therapy [13]. Mathematical modeling studies based on data from sub-Saharan African countries have suggested that substantial declines in tuberculosis incidence could be achieved by initiating antiretroviral therapy as soon as HIV infection is known, without waiting for a threshold CD4 count to be reached [21], [22]. Thus, further expansion of HIV testing initiatives and earlier initiation of antiretroviral therapy will be necessary to realize the full benefit of antiretroviral therapy on reducing tuberculosis incidence in Kenya.

While continued scale-up of antiretroviral therapy programs is important, multiple studies have suggested that tuberculosis incidence among persons on antiretroviral therapy and with high CD4 counts still exceeds incidence in the HIV-negative population [23]–[25]. Therefore, additional interventions will be necessary if the disparity in tuberculosis incidence between persons with and without HIV is to be addressed. These include identification of all persons living with HIV, early initiation of antiretroviral therapy, routine symptom-based screening for tuberculosis disease, and provision of tuberculosis preventive therapy to prevent development of tuberculosis disease.

World Health Organization guidelines recommend routine symptom-based screening for tuberculosis among persons living with HIV and provision of tuberculosis preventive therapy for persons with a negative symptom screen [26]. In persons with HIV who have a positive tuberculin skin test result, tuberculosis preventive therapy can reduce the risk of developing tuberculosis disease by over 60% [7] and the combination of antiretroviral therapy and continuous tuberculosis preventive therapy can reduce tuberculosis incidence by over 90% [8]. While screening for tuberculosis has been integrated into HIV care in Kenya, tuberculosis preventive therapy has not yet been widely implemented [10]. To achieve the maximum benefit of Kenya’s integrated TB/HIV policy, it is important that screening for tuberculosis among persons living with HIV be rigorous, that every person meeting criteria for further evaluation be tested with a sensitive tuberculosis diagnostic test, and that if indicated, preventive therapy be given to those confirmed not to have tuberculosis.

Although tuberculosis disproportionately affects persons living with HIV, much transmission of tuberculosis is driven by persons without HIV, who typically remain contagious for longer periods of time [27]. Therefore, interventions to decrease tuberculosis incidence in the general population are necessary to prevent new infections among persons living with HIV. As delayed diagnosis leads to prolongation of infectiousness and effective treatment rapidly attenuates infectiousness [17], [28], early diagnosis and initiation of appropriate treatment are critical to reducing transmission. Accomplishing both of these goals will require diagnostic tests more sensitive than sputum-smear microscopy and increasing early tuberculosis case finding through active case finding strategies.

Interventions aimed at reducing transmission of HIV may also have the indirect benefit of reducing tuberculosis incidence. Our crude analysis suggests that during 1998–2007, the proportion of tuberculosis cases attributable to HIV declined by roughly 50%, driven by a decline of similar magnitude in HIV prevalence in the overall population. Thus, further reductions in HIV transmission could decrease the pool of vulnerable persons who are at higher risk of developing tuberculosis, which in turn should decrease new tuberculosis cases.

Our analysis was subject to several limitations. The proportion of tuberculosis patients with unknown HIV status, particularly during early years of the analysis period, prevented us from making point estimates of incidence. In addition, because tuberculosis case data were incompletely disaggregated by age, we assumed a negligible number of tuberculosis patients over 64 years of age to match the age group to which the KAIS estimates correspond. However, sensitivity analyses suggested that making different assumptions based on the age distribution of adult tuberculosis patients in districts where more detailed data were available would have changed our estimates by less than 10%, and the same trends would have been observed.

The lack of recorded HIV test results for tuberculosis cases reported before 2006 forced us to make crude estimates to analyze trends in tuberculosis incidence before 2006. These crude estimates relied on the assumption of a constant prevalence ratio of HIV infection between tuberculosis patients and the general population when this ratio may in fact have changed over time. Furthermore, the prevalence of HIV infection used in the crude estimates came from a mathematical model based on HIV prevalence data from anonymous serosurveys among pregnant women attending public antenatal clinics, although the projection curves for the model were calibrated using national estimates of HIV prevalence from historical population-based household surveys [15].

Finally, our model did not take into account changes in tuberculosis case detection rate or treatment success rate over time. Although programmatic estimates suggest case detection rates between 70–85% starting in 1998 [14], inaccuracy in these estimates would be propagated in our incidence estimates. If case detection rates in the early part of the analytic period were in fact lower than estimated, then tuberculosis incidence may have been even higher during that period, and the decline in incidence consequently sharper. Treatment success rates were high throughout the study period (81–88% for new sputum smear-positive cases [29], [30]), so changes in treatment success rate were unlikely to have affected tuberculosis incidence.

In conclusion, the decline in tuberculosis incidence both among adults living with HIV and adults without HIV suggests the success of tuberculosis control efforts in Kenya in recent years and likely the effect of expanded HIV testing and antiretroviral therapy programs. However, the persistent high incidence of tuberculosis among adults living with HIV is cause for concern, given that tuberculosis remains the leading cause of death in people with HIV. A concerted effort aimed at decreasing transmission by controlling tuberculosis in the general population should be combined with specific interventions among persons living with HIV to reduce tuberculosis incidence among this population.

## Disclaimer

The views and opinions expressed in this article are those of the authors and do not necessarily represent an official position of the U.S. Centers for Disease Control and Prevention.
